# Correlation between epicardial adipose tissue and cognitive performance in older adults. A role for autonomic nervous system imbalance?

**DOI:** 10.3389/fragi.2026.1711862

**Published:** 2026-03-26

**Authors:** Martina Valentino, Maria Perrotti, Mariapia Calligari, Luca Corbia, Carmine Dello Russo, Roberto Vicidomini, Stefano Maisto, Francesco De Martino, Marta Zuccarino, Natascia De Lucia, Valentina Parisi, Maddalena Conte, Klara Komici, Giuseppe Rengo, Dario Leosco, Grazia Daniela Femminella, Laura Petraglia

**Affiliations:** 1 Department of Translational Medical Sciences, University of Naples Federico II, Naples, Italy; 2 Department of Brain Sciences, Imperial College London, London, United Kingdom; 3 Department of Neurosciences, Reproductive and Odontostomatological Sciences, University of Naples Federico II, Naples, Italy; 4 Department of Medicine and Health Sciences “V. Tiberio”, University of Molise, Campobasso, Italy; 5 Istituti Clinici Scientifici Maugeri IRCCS-Scientific Institute of Telese Terme, Telese Terme, Benevento, Italy; 6 ANASTE Humanitas Foundation, Roma, Italy

**Keywords:** autonomic nervous system function, cognitive function, epicardial adipose tissue, heart rate variability, mild cognitive impairment, mini mental state examination

## Abstract

**Background:**

Epicardial Adipose Tissue (EAT) is the visceral fat depot of the heart and evidence suggests that increased EAT thickness is associated with reduced cognitive performance in older adults, although the exact mechanisms remain poorly defined. Heart Rate Variability (HRV), an indicator of autonomic nervous system (ANS) activity, has been linked to both EAT volume and cognitive decline. The aim of this study was to assess the association between EAT thickness and cognitive status in an older population. Further, we explored, through HRV metrics, the ANS as a potential link for this association.

**Methods:**

99 participants aged over 65 were enrolled at the outpatient clinics of the Federico II University Hospital. Participants underwent comprehensive medical and cardiological evaluations, including transthoracic echocardiography, 24 h Holter electrocardiographic (ECG) monitoring, and cognitive assessment using the Mini-Mental State Examination (MMSE).

**Results:**

A significant inverse correlation was observed between EAT thickness and MMSE scores (ρ = −0.341, p = 0.001), while EAT thickness showed a positive association with the standard deviation of normal-to-normal intervals (SDNN) index of HRV (ρ = 0.212, p = 0.036). EAT thickness was greater in individuals with dementia compared to cognitively normal subjects, whereas the low frequency (LF)/high frequency (HF) ratio was significantly reduced in the dementia group respect to those with mild cognitive impairment (MCI). Multivariate analysis showed that EAT thickness was an independent predictor of MMSE scores after adjusting for age, sex, body mass index, comorbid conditions, SDNN index and LF/HF.

**Conclusion:**

Our findings indicate that greater EAT thickness is independently associated with poorer cognitive performance in older adults and may reflect both increased visceral fat burden and underlying inflammatory processes. Further studies on larger cohorts are needed to clarify the mechanisms underlying the EAT-autonomic-cognition axis.

## Introduction

Epicardial Adipose Tissue (EAT) represents the cardiac visceral fat deposit, located between the myocardium and the visceral layer of the pericardium. Under physiological conditions, it has a thermogenic action, provides mechanical protection and is responsible for the release of anti-inflammatory cytokines. Several pieces of evidence suggest that increased EAT thickness is associated with unfavourable anatomical and electrical remodelling of the heart related to an increased and sustained inflammatory stimulus and oxidative stress (Iacobellis, 2022). It secretes various pro-inflammatory and pro-atherogenic cytokines, such as Tumor Necrosis Factor-α (TNF-α), Interleukin-6 (IL-6), and Interleukin-8 (IL-8), which may promote the development of cardiovascular diseases (Iacobellis, 2022; Packer, 2018).

EAT thickness, as assessed by transthoracic echocardiography, has been established as a non-invasive and clinically useful marker of visceral adiposity and cardiovascular risk. Beyond its cardiovascular involvement, EAT has also been implicated in neuroinflammatory processes. Experimental studies have demonstrated that cytokines derived from adipose tissue can impair memory function in animal models of obesity (Bray, 2014; Butler et al., 2025). In particular, elevated levels of Interleukin-1β (IL-1β) have been associated with increased adiposity and cognitive decline (Bray, 2014). In healthy older adults, greater EAT thickness has been correlated with poorer cognitive performance (Mazzoccoli et al., 2014); however, the biological mechanisms linking epicardial fat accumulation and neurocognitive function remain largely unexplored.

Heart rate variability (HRV) reflects the functional integrity of the autonomic nervous system and is defined as the physiological fluctuation in the time intervals between consecutive heartbeats. HRV can be reliably assessed through 24 h electrocardiographic (ECG) monitoring, which captures autonomic responses during routine daily activities. This long-term monitoring approach is particularly valuable for risk stratification across various clinical conditions and for detecting subclinical autonomic dysfunction (Shaffer and Ginsberg, 2017; Kleiger et al., 2005; Quigley et al., 2024). In healthy individuals, a balanced sympathovagal tone ensures adequate self-regulation and adaptability of cardiovascular function. Conversely, disruption of this balance is often regarded as an early indicator of cardiac autonomic neuropathy and a potential contributor to cardiovascular morbidity. In this context, a relative predominance of sympathetic activity or a reduction in parasympathetic modulation is generally interpreted as reflecting poorer autonomic function, whereas preserved or dominant parasympathetic modulation is considered more favorable, although the interpretation of sympathovagal balance indices remains debated and not univocal (Shaffer and Ginsberg, 2017; Kleiger et al., 2005).

Altered HRV, particularly reflecting diminished parasympathetic modulation, has been observed in individuals with mild cognitive impairment (MCI) (Collins et al., 2012). Notably, EAT contains intrinsic cardiac ganglia and autonomic nerve fibers, facilitating direct interaction with the autonomic nervous system (White, 2016; Balcioğlu et al., 2015; Parisi et al., 2016). It has been reported an arrhythmogenic role of EAT, particularly in the pathogenesis of atrial fibrillation (Conte et al., 2022). Moreover, recent studies have demonstrated a link between increased EAT thickness and autonomic imbalance, as evidenced by HRV indices (Balcioğlu et al., 2015). Experimental animal models further support this association; mice lacking EAT exhibit autonomic dysregulation characterized by sympathetic dominance (Carnevali et al., 2014).

The present study aims to investigate the association between EAT thickness and cognitive performance in older adults, and to explore the potential mediating role of autonomic nervous system activity as assessed by HRV parameters.

## Methods

### Study population

Ninety-nine subjects, older than 65 years, were enrolled at the outpatient clinics of the Geriatrics Unit of the Federico II University Hospital of Naples from May 2023 to November 2024. All patients had undergone a complete medical history collection, accurate clinical and cardiological examinations, performed a transthoracic echocardiography and a 24 h ECG analysis recording. Volunteers were included if they were 65 and older, cognitively normal or with a diagnosis of minor or major neurocognitive disorder. For the assessment of cognitive function, patients underwent a Mini Mental State Examination (MMSE). Cognitively normal subjects were individuals with no cognitive concern (from patient and informant) and a MMSE score ≥28/30. Subjects with MCI and mild/moderate dementia had the diagnosis made according to the DSM-V criteria for minor and major neurocognitive disorders, respectively, and MMSE scores comprised between 24/30 and 27/30 for MCI and between 14/30 and 23/30 for dementia.

Exclusion criteria were as follows: atrial fibrillation, pacemaker/defibrillator, use of antipsychotics, severe depression, ECG Holter recording <20 h duration, high chest acoustic impedance, inability to provide informed consent.

The decision to include only patients with a sinus rhythm and to exclude those with atrial fibrillation, pacemakers or defibrillators was made to ensure the accuracy of HRV measurements, which are unreliable in the presence of irregular rhythms or artificial pacing. Similarly, patients on antipsychotic medications or with severe depressive symptoms were excluded to avoid confounding factors known to independently affect both autonomic function and cognitive performance. The exclusion of subjects with previous major cardiovascular events, such as myocardial infarction, was also necessary to isolate the effects of EAT thickness from overt structural heart disease and its direct impact on both HRV and cognition. Finally, the requirement of high-quality, ≥20 h Holter recordings was essential to ensure the reliability of HRV parameters, which are susceptible to artifacts and signal interruptions.

The study was conducted in accordance with the Ethical standards of Helsinki Declaration, and the research protocol was reviewed and approved by the Local Ethics Committee (protocol number 330/2024).

### Epicardial adipose tissue measurement

A complete echocardiographic examination was performed with GE Healthcare Vivid E9 machine.

EAT was measured with patients in the left lateral decubitus position, using a high-resolution phased-array transducer, in the parasternal long-axis view at the level of the Rindfleisch fold, a recognized pericardial recess located between the aortic root and the right ventricular outflow tract (Parisi et al., 2020).

Cardiac cycles were synchronized using continuous ECG monitoring to ensure consistent acquisition timing. Measurements were obtained at end-systole, when the heart is maximally contracted and the EAT deposition is most clearly delineated. Three cardiac cycles were analysed, and the average value was used for statistical purposes. Care was taken to avoid inclusion of pericardial layers or fluid collections. All scans were reviewed offline by two independent operators blinded to the clinical data, and intra- and inter-observer variability were minimized through standardized training and protocol adherence.

### Heart rate variability measurement

24 h Holter ECG recordings were obtained using three-channel digital recorders (DMS 300-4L/AL). Recordings of at least 20 h and of sufficient quality were included in the analysis. Evaluations were performed by two experienced clinicians blind to the MMSE scores. The ECG data were manually preprocessed to visually verify all complexes automatically marked as atrial and ventricular ectopic beats. The following time domain HRV indexes were derived using the DMS CardioScan (Version 12.5.0079a): square root of the mean squared differences of successive normal-to-normal (NN) intervals (RMSSD), the standard deviation (SD) of all NN intervals (SDNN), the mean of the deviation of the 5 min NN intervals over the entire recording (SDNN index), the SD of the average NN intervals for each of the 5 min segments of the entire recording (SDANN), and the proportion of adjacent RR intervals differing by *>*50 m in the 24 h recording (pNN50) were measured. Additionally, the following frequency domain indexes were calculated: the power in low frequency (LF) range (0·04–0·15 Hz), the power in high frequency (HF) range (0·15–0·4 Hz) and their ratio (LF/HF).

All measurements were performed according to the Task Force of the European Society of Cardiology and the North American Society of Pacing and Electrophysiology (Heart rate variability, 1996).

### Statistical analysis

Continuous variables were presented as mean ± SD and nominal variables were presented as number of cases with percentage. Normal distribution was tested using the Shapiro-Wilk test. The *χ*2 test was used for comparison of categorical variables between two groups. Continuous variables were compared using the Student’s t-test and the Mann-Whitney U-test where appropriate. The correlation between MMSE scores, the Holter measures and EAT thickness were analyzed using the Spearman’s test. Multiple linear regression models were built to evaluate the independent associations of MMSE and EAT, correcting for age and gender, as well as for body mass index (BMI), comorbidities (diabetes, dyslipidaemia, hypertension) and HRV parameters found to be significant at univariate correlations or at group comparisons. The Statistical Program for Social Sciences (SPSS for Windows 29, IBM Corp.) was used for all statistical calculations. Mediation analysis was conducted using the Process macro 5.0 in SPSS, with a confidence level of 95 and 5,000 bootstrap samples. The independent variable (X) was EAT, while the dependent variable (Y) was MMSE. Model 4 in the Process macro was used to analyze the direct and indirect effects of EAT on MMSE, with SDNN and LF/HF as mediators. All tests of significance were two-tailed and statistical significance was defined as p *<* 0.05. The graphs were prepared with *jamovi* (Version 2.6, https://www.jamovi.org).

## Results

The description of all the characteristics of the study population is represented in Table 1. The mean age of the enrolled subjects was 75.5 ± 6.8 years, with a male/female ratio 53/46. The mean BMI was 25.9 ± 4.6 kg/m2. The mean EAT thickness was 14.3 ± 3.7 mm and the mean MMSE scores were 25.9 ± 3.7. 68 subjects (69%) suffered from hypertension, 24 from diabetes (24%) and 63 from dyslipidemia (64%). A small proportion (15%) had carotid plaques. In the study population, 41 subjects were cognitively normal (CN), 38 had a diagnosis of MCI and 20 suffered from mild or moderate dementia.

**TABLE 1:** Characteristics of the study population.

Age, years	75.5 ± 6.8
Sex (M/F)	53/46
Diagnostic group (CN/MCI/Dementia)	41/38/20
MMSE	25.9 ± 3.7
BMI (kg/m^2^)	25.9 ± 4.6
EAT thickness, mm	14.3 ± 3.7
pnn50, %	15.5 ± 17.5
rMSSD, ms	47.4 ± 36.8
SDNN, ms	126.3 ± 43.0
SDANN, ms	108.2 ± 37.8
SDNN index, ms	71.5 ± 153.3
LF, ms^2^	660.0 ± 875.8
HF, ms^2^	351.2 ± 744.9
LF/HF	2.6 ± 1.5
Hypertension (YES/NO)	68/31
Diabetes (YES/NO)	24/75
Dyslipidaemia (YES/NO)	63/36
Carotid plaques (YES/NO)	15/84

Continuous data are presented as mean ± SD.

CN, cognitively normal; MCI, mild cognitive impairment; EAT, epicardial adipose tissue; pnn50, Percentage of successive normal-to-normal intervals that differ by more than 50 milliseconds; rMSSD, root mean square of the successive differences; SDNN, Standard Deviation of All Normal-to-Normal Intervals; SDANN, Standard Deviation of the Average NN, Intervals in 5-Minute Segments; SDNN, index, Mean of the Standard Deviations of NN, Intervals in All 5-Minute Segments; LF, low frequency power; HF, high frequency power; LF/HF, ratio of low frequency to high frequency power.

**FIGURE 1:**
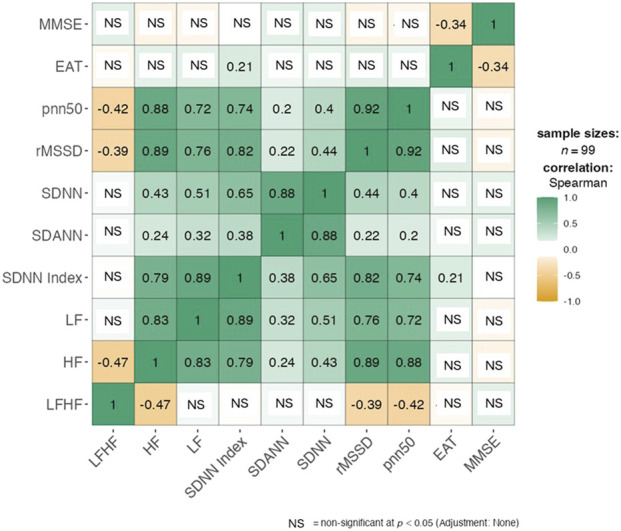
Spearman correlation analysis. Significant inverse correlation between the EAT thickness and MMSE scores (ρ = −0.341, p = 0.001) Significant direct correlation between EAT thickness and the SDNN index of the HRV (ρ = 0.212, p = 0.036). No significant associations were detected between MMSE scores and either time domain or frequency domain indexed of the HRV.

The results of the Spearman correlation analysis are shown in Figure 1. There was a significant inverse correlation between the EAT thickness and MMSE scores (ρ = −0.341, p = 0.001), and a significant direct correlation between EAT thickness and the SDNN index of the HRV (ρ = 0.212, p = 0.036). No significant associations were detected between MMSE scores and either time domain or frequency domain indexed of the HRV in this population.

**FIGURE 2:**
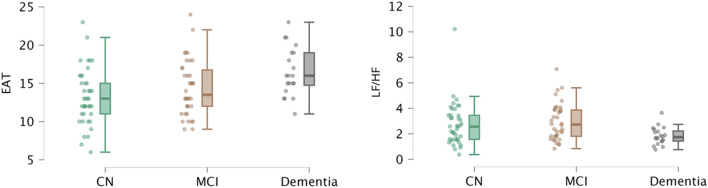
EAT thickness and LF/HF ratio in the study population. EAT thickness (in mm) was significantly higher in the dementia group compared to the cognitively normal subjects (CN). The LF/HF ratio was significantly lower in the dementia group compared to the mild cognitive impairment (MCI) group. The three groups are defined in the Methods section.

When stratifying the population according to the diagnostic group to compare EAT thickness and HRV indexes, we found that EAT thickness was significantly higher in the dementia group compared to the CN, and that the LF/HF ratio was significantly lower in the dementia group compared to the MCI group (Figure 2; Supplementary Table S1).

To evaluate the independent predictors of cognitive function in this population, we built regression models with MMSE scores as the dependent variable, corrected by age and gender, as shown in Table 2. In model 1 (F(3,95) = 7.431, p < 0.001, *R* ^2^ = 0.19)), EAT and age showed a significant independent predictive value (for EAT β = −0.233, t(96) = -2.493, p = 0.014). In model 2, we added BMI values, diabetes, hypertension and dyslipidemia as predictors; the model proved significant (F(7,86) = 2.333, p = 0.03, *R* ^2^ = 0.16)), with EAT and age showing independent predictive values on MMSE scores (for EAT: β = −0.237, t(91) = -2.232, p = 0.028; for age: β = −0.301, t(91) = −2.918, p = 0.004). Finally, in model 3 we added SDNN index and LF/HF ratio as independent predictors of MMSE scores; the model proved significant (F(9,84) = 2.218, p = 0.029, *R* ^2^ = 0.19)), with EAT and age showing independent predictive values (for EAT: β = −0.233, t(89) = -2192, p = 0.031; for age: β = −0.267, t(89) = -2.567, p = 0.012).

**TABLE 2:** Multiple regression models for MMSE scores in the study population.

Model 1	Unstandardized coefficients		Standardized coefficients	t	Sig
	B	Std. Error	Beta		
(Constant)	43.875	4.103	​	10.694	0.000
Sex	−0.575	0.687	−0.078	−0.837	0.405
Age	−0.183	0.051	−0.333	−3.565	**0.001**
EAT	−0.233	0.094	−0.233	−2.493	**0.014**
Model summary: (F(3,95) = 7.431, p < 0.001, *R* ^2^ = 0.19					

Model 2	Unstandardized coefficients		Standardized coefficients	t	Sig
	B	Sth. Error	Beta		
(Constant)	40.049	4.696	​	8.528	0.000
Sex	−0.517	0.713	−0.073	−0.724	0.471
Age	−0.160	0.055	−0.301	−2.918	**0.004**
EAT	−0.229	0.102	−0.237	−2.232	**0.028**
Dislipidemia	−0.198	0.794	−0.027	−0.250	0.803
Hypertension	0.258	0.796	0.034	0.324	0.747
Diabetes	−0.408	0.848	−0.050	−0.481	0.631
BMI	0.082	0.083	0.106	0.984	0.328
Model summary: F(7,86) = 2.333, p = 0.03, *R* ^2^ = 0.16					

Model 3	Unstandardized coefficients		Standardized coefficients	t	Sig
	B	Sth. Error	Beta		
(Constant)	37.064	4.949	​	7.489	0.000
Sex	−0.194	0.736	−0.027	−0.263	0.793
Age	−0.142	0.055	−0.267	−2.567	**0.012**
EAT	−0.225	0.103	−0.233	−2.192	**0.031**
Dyslipidemia	−0.138	0.789	−0.019	−0.174	0.862
Hypertension	0.693	0.824	0.090	0.840	0.403
Diabetes	−0.778	0.881	−0.096	−0.883	0.380
BMI	0.069	0.083	0.090	0.832	0.408
SDNN index	−0.001	0.002	−0.048	−0.474	0.636
LF/HF	0.478	0.265	0.202	1.808	0.074
Model summary: F(9,84) = 2.218, p = 0.029, *R* ^2^ = 0.19					

Significant predictors are in bold.

EAT, epicardial adipose tissue; BMI, body mass index; SDNN, index, Mean of the Standard Deviations of NN, Intervals in All 5-Minute Segments; LF/HF, ratio of low frequency to high frequency power.

**FIGURE 3:**
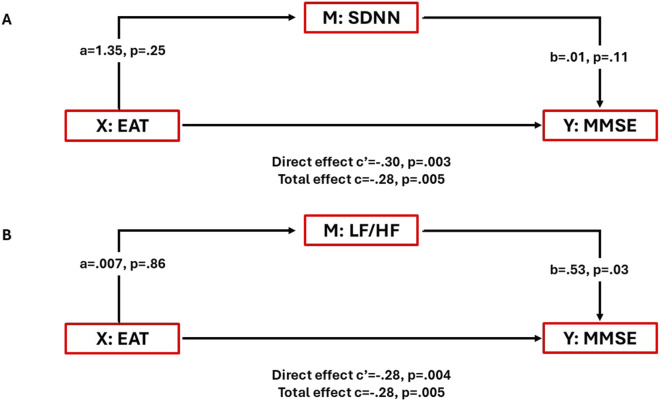
Mediation analysis of SDNN and LF/HF between EAT and MMSE. **(A)** Although the direct effect of EAT on MMSE scores was significant (total effect β = −0.28, p = 0.005, 95% CI -0.4745, −0.0866), SDNN did not show a mediating effect on this association. **(B)** LF/HF is associated with MMSE scores (β = 0.53, p = 0.03), but it does not mediate the EAT-MMSE association.

Moreover, to test the possible effect of HRV measures on the association between EAT and MMSE in this population, we carried out a mediation analysis with SDNN and LF/HF as mediators. The direct effect of EAT on MMSE scores was significant in both models. While SDNN did not show a mediating effect on this association, LF/HF was associated with cognition (β = 0.53, p = 0.03) but did not have a mediating effect on the EAT-MMSE relationship (Figure 3).

## Discussion

This study demonstrates a significant inverse relationship between EAT thickness and cognitive performance in older adults. Notably, EAT thickness emerged as an independent predictor of MMSE scores across all multivariate regression models, even after adjusting for major confounders such as age, sex, BMI, and cardiovascular risk factors. Moreover, while no significant correlations were observed between MMSE scores and most HRV indices, EAT thickness was directly associated with the SDNN index. When stratified by cognitive status, individuals with dementia exhibited significantly higher EAT thickness compared to cognitively normal subjects, and a significantly lower LF/HF ratio compared to the MCI group, reinforcing the hypothesis of a link between altered autonomic regulation and cognitive impairment. Indeed, our mediation analysis has shown a direct effect of LF/HF on MMSE scores, suggesting that autonomic balance may relate to cognition, but it is not the pathway linking EAT to cognition.

Our findings are in accordance with previous evidence supporting the role of EAT not only in the development of cardiovascular diseases, but also in neurocognitive decline. EAT is a distinct visceral fat depot located between the myocardium and the visceral layer of the pericardium, preferentially accumulating along the atrioventricular and interventricular grooves and surrounding the coronary arteries. This depot is biologically distinct from pericardial and subcutaneous adipose tissue and is characterized by specific metabolic, inflammatory, and neurohumoral properties (Iacobellis, 2022; Packer, 2018). Under normal physiological conditions, EAT has thermogenic and protective mechanical properties and plays a role in modulating local inflammation. However, when increased in thickness, EAT becomes a significant source of pro-inflammatory and pro-atherogenic cytokines, such as IL-6, IL-8, TNF-α, and IL-1β, that are capable of promoting and perpetuating systemic inflammation, oxidative stress, and endothelial dysfunction (Iacobellis, 2022; Packer, 2018; Oikonomou and Antoniades, 2019).

Neuroinflammatory mechanisms are increasingly recognized as contributors to cognitive impairment and dementia. Experimental studies in obese animal models have shown that adipose tissue–derived cytokines can cross the blood-brain barrier, activate microglia, and impair hippocampal function (Erion et al., 2014). In human studies, elevated systemic levels of IL-1β and other inflammatory markers have been associated with both increased adiposity and lower cognitive performance (Miller and Spencer, 2014). These observations provide a biological framework linking adiposity, inflammation, and cognitive decline. EAT, given its proximity to the coronary circulation and rich vascular and neuronal supply, may act as a key mediator of these systemic effects, thus representing a possible cardio-cerebral risk link (Iacobellis, 2022; Packer, 2018); however, in the present study no inflammatory biomarkers were assessed, and therefore any inference regarding neuroinflammatory mechanisms should be considered speculative.

In this study HRV indices were evaluated to explore their potential mediating role in the relationship between EAT thickness and cognitive performance. SDNN, is a time-domain measure, representing the standard deviation of all normal-to-normal (NN) intervals over a 24 h period, and has been widely validated as a robust index of global autonomic modulation (Shaffer and Ginsberg, 2017; Kleiger et al., 2005). Thus, SDNN reflects overall heart rate variability over a 24 h period and integrates mixed autonomic influences across circadian rhythms and daily activities, whereas other HRV indices such as RMSSD and pNN50 predominantly capture short-term parasympathetic modulation, and SDANN emphasizes longer-term components of variability. Lower SDNN values are consistently associated with increased cardiovascular risk and impaired parasympathetic activity (Heart rate variability, 1996). Several studies have also linked decreased SDNN to cognitive dysfunction, particularly in older adults and individuals with MCI. Similarly, the LF/HF ratio, is a frequency-domain parameter traditionally interpreted as a marker of sympathovagal balance, which reflects the relative contribution of sympathetic and parasympathetic nervous system activity, and has been found to be altered in patients with neurodegenerative disorders. A lower LF/HF ratio, indicative of reduced sympathetic modulation or increased parasympathetic tone, has been observed in MCI and dementia, although its interpretation remains complex and not well understood (Sandler et al., 2021; Cheng et al., 2022; Schaich et al., 2020; Nicolini et al., 2020).

Emerging evidence also supports a relationship between increased EAT thickness and autonomic dysregulation, as measured by HRV. EAT is richly innervated by autonomic nerve fibers and contains intrinsic cardiac ganglia, enabling it to influence cardiac autonomic control directly (White, 2016; Balcioğlu et al., 2015; Parisi et al., 2016). Importantly, previous work from our group based on epicardial fat biopsies obtained at the level of the Rindfleisch fold demonstrated increased local catecholaminergic activity compared with subcutaneous adipose tissue, supporting the biological plausibility of a link between EAT and cardiac autonomic regulation (Parisi et al., 2016). Since this prior evidence, we hypothesized that increased EAT thickness might be associated with alterations in HRV indices. However, we acknowledge that enhanced local catecholaminergic activity within EAT does not necessarily translate into overt or consistent changes in global HRV measures, which reflect complex and integrated autonomic regulation at the systemic level. In our study, EAT thickness was independently associated with the SDNN index, suggesting a possible link between visceral adiposity and altered autonomic tone. Although higher SDNN values are commonly considered favorable in cardiovascular populations, interpretation of HRV metrics in older adults and in the context of neurocognitive disorders is more complex. In such settings, increased variability may reflect altered or compensatory autonomic responses rather than preserved autonomic function. In the present study, the association between EAT thickness and SDNN index was modest and did not remain independently associated with cognitive performance. Furthermore, individuals with dementia demonstrated a significantly lower LF/HF ratio compared to those with MCI, reinforcing the hypothesis that autonomic imbalance may contribute to cognitive decline. This apparent dissociation supports a cautious interpretation of HRV findings and suggests that increased EAT may be linked to autonomic dysregulation rather than to a beneficial autonomic profile.

Taken together, these findings align with previous experimental and clinical studies indicating that visceral fat depots, particularly EAT, may be associated with neurocognitive impairment, potentially through inflammatory and autonomic pathways described in prior literature.

This study strengthens the hypothesis that EAT is not merely a cardiovascular risk marker but may also be involved in the pathogenesis of cognitive decline. EAT thickness, assessed non-invasively by transthoracic echocardiography, could represent a biomarker for early identification of individuals at risk for cognitive impairment. Future research should further explore the interplay between EAT, HRV, and cognitive trajectories, ideally incorporating longitudinal designs and complementary biomarkers of systemic inflammation and neural integrity. Moreover, larger studies are warranted to clarify the causality of these associations and to explore whether targeted reduction of EAT, through lifestyle or pharmacologic interventions, may have a protective effect on cognitive aging.

### Limitations

This study presents several limitations. Cognitive function was assessed using only the MMSE, which, although widely used, may not capture subtle deficits in specific cognitive domains. The absence of inflammatory biomarkers and neuroimaging data limits the ability to directly investigate neuroinflammatory mechanisms underlying the observed associations. Levels of physical activity, which can influence both autonomic function and adiposity, were not measured. Other potential confounders such as sleep quality and general anxiety levels were not measured in this study and might have improved the explanatory power of our model. While 24 h Holter ECG is a validated method for assessing heart rate variability (HRV), short-term HRV recordings, which are also used in clinical and research settings, were not included, preventing comparison across time scales. Moreover, daytime and nighttime HRV measures were not compared on this population, and it might be useful to explore any potential differences in future work. Although prior evidence supports a link between epicardial adipose tissue and cardiac autonomic regulation, HRV indices reflect integrated systemic autonomic function and may not fully capture the effects of local catecholaminergic activity within EAT. In addition, epicardial adipose tissue was assessed using a single-point echocardiographic thickness measurement, which, although standardized and reproducible, does not capture the full three-dimensional volume or regional heterogeneity of epicardial fat compared with multi-site or volumetric imaging techniques. Finally, the cross-sectional nature of the study limits conclusions about causality, and findings need to be confirmed in larger, longitudinal cohorts.

## Conclusion

Our findings support the emerging role of EAT as a modifiable risk factor with relevance beyond cardiovascular health, potentially extending to neurocognitive domains. Future studies incorporating more comprehensive neuropsychological batteries, inflammatory markers, and longitudinal follow-up are needed to clarify causality and evaluate the impact of targeted interventions on cognitive outcomes.

## Data Availability

The raw data supporting the conclusions of this article will be made available by the authors, without undue reservation.
