# Supplementary material for: Correlation between epicardial adipose tissue and cognitive performance in older adults. A role for autonomic nervous system imbalance?
Source: Front Aging. 2026 Mar 26;7:1711862. doi: 10.3389/fragi.2026.1711862 (PMC13061875; doi:10.3389/fragi.2026.1711862)
Supplement: Supplementary file 1 [file Table1.docx]

**Supplemental Table 1. HRV measures across cognitive function groups**

|  | **CN** | **MCI** | **Dementia** |
| --- | --- | --- | --- |
| pnn50, % | 12.3±12.6 | 15.6±18.1 | 21.8±23.4 |
| rMSSD, ms | 43.4±35.0 | 46.5±34.0 | 57.3±44.9 |
| SDNN, ms | 127.5±35.0 | 131.7±40.8 | 113.8±59.2 |
| SDANN, ms | 110.7±31.9 | 111.5±40.6 | 96.7±43.0 |
| SDNN Index, ms | 52.8±24.4 | 96.7±24.2 | 62.1±40.8 |
| LF, ms^2^ | 478.2±437.2 | 678.6±717.5 | 997.5±1536.0 |
| HF, ms^2^ | 229.8±226.8 | 272.8±311.8 | 749.2±1534.0* |
| LF/HF | 2.7±1.7 | 2.9±1.4 | 1.8±0.7# |

Data are presented as mean ±SD; *p<.05 vs CN; #p<.05 vs MCI
